# Inhibition of both endplate nutritional pathways results in intervertebral disc degeneration in a goat model

**DOI:** 10.1186/s13018-019-1188-8

**Published:** 2019-05-16

**Authors:** Si Yin, Heng Du, Weigong Zhao, Shaohui Ma, Ming Zhang, Min Guan, Miao Liu

**Affiliations:** 1 Department of Orthopaedic Surgery, First Affiliated Hospital of Xi’an Jiaotong University, Room 1501, Inpatient Building, No. 277, Road Yantawest, Xi’an, 710061 Shaanxi Province China; 2 Department of Medical Imaging, First Affiliated Hospital of Xi’an Jiaotong University, Xi’an, 710061 Shaanxi Province China; 3 Department of Medical Imaging, Henan Provincial People’s Hospital, Zhengzhou, 450003 Henan Province China

**Keywords:** Endplate nutritional pathway, Intervertebral disc degeneration, Bone cement, Goat model

## Abstract

**Background:**

The vertebral endplate route was demonstrated to be the main pathway for nutrition to the intervertebral disc. However, it is still a controversial issue on whether the blocking of the endplate nutritional pathway could result in intervertebral disc degeneration (IDD) in animal models. The aim was therefore to investigate the effect of the inhibition of both endplate nutritional pathways by bone cement injection on the IDD in a goat model.

**Methods:**

Two lumbar intervertebral discs (L2–3 and L3–4) in eight 24-month-old goats were blocked in both endplate nutritional pathways by cement injection, and the other two lumbar intervertebral discs (L1–2 and L4–5) remained intact as normal controls. Effective blocking area percentage in nucleus pulposus (NP) was calculated, and X-rays, magnetic resonance imaging (MRI), and histology studies were performed at 4, 12, 24, and 48 weeks after operation.

**Results:**

The mean effective blocking area percentage was 60.7 ± 5.3%. Imaging examinations at the time of 48 weeks after blocking the endplate nutritional pathways showed obvious IDD, with larger disc height reduction and higher degrees of disc degeneration grading compared with the normal controls. Histological examinations including HE, Masson’s trichrome, Sirius Red, and proteoglycan stainings also confirmed the degenerative changes of the blocked discs.

**Conclusions:**

The endplate nutritional route could be inhibited by blocking both endplate pathways with cement injection in a goat model. The severe inhibition in the endplate nutritional pathways may result in IDD.

## Background

Many factors are considered to be relevant with intervertebral disc degeneration (IDD), such as reduced nutrition supply, imbalanced proliferation and apoptosis of nucleus pulposus cells, loss of proteoglycan and water content in extracellular matrix, repeated mechanical stress, and abnormal autoimmune response [1]. The nutrition supply is perceived as one of the most important factors in maintaining the biological function of intervertebral disc, which obtain nutrition from the blood vessels in the adjacent vertebrae by a process of diffusion through the endplate as the main pathway [2].

Numerous studies have indicated that the reduction of nutrition supply in intervertebral disc could lead to the decrease of the concentration and biological activities of nucleus pulposus cells (NPCs), and even the development of IDD [3, 4]. Thus, it has been hypothesized that if the endplate nutritional pathway is blocked, there might be the development of nutrient deficiency for the intervertebral disc, which might result in IDD. However, the hypothesis is still controversial in animal models. Hutton et al. [5] blocked an endplate nutritional pathway with bone cement in the dog model for up to 70 weeks and did not produce obvious degeneration in the intervertebral disc. Meanwhile, Kang et al. [6] demonstrated that blocking both endplate pathways in an immature porcine model could cause IDD after 3 months of bone cement intervention. We therefore decided to carry out this study to demonstrate the effect of the inhibition of both endplate nutritional pathways by bone cement injection on the IDD in a goat model, in order to clarify the previously ambiguous findings.

## Methods

### Animals

Eight Chinese Guanzhong mature goats (all females, 24 months old, weight range from 35 to 45 kg, average weight 41.3 ± 5.1 kg) from the same pasturing area were provided by the Animal Center of the Medical School of Xi’an Jiaotong University for the current study. The animal experiments were approved by the Animal Ethics Sub-Committee of the First Affiliated Hospital of Xi’an Jiaotong University. After standardized feeding for 2 weeks, all the goats were sedated and anesthetized with Su-Mian-Xin II (intramuscular injection, 0.3 ml/kg) and propofol (intravenous drip, 2 mg/kg) before the surgical procedure.

### Surgical procedure

**Fig. 1:**
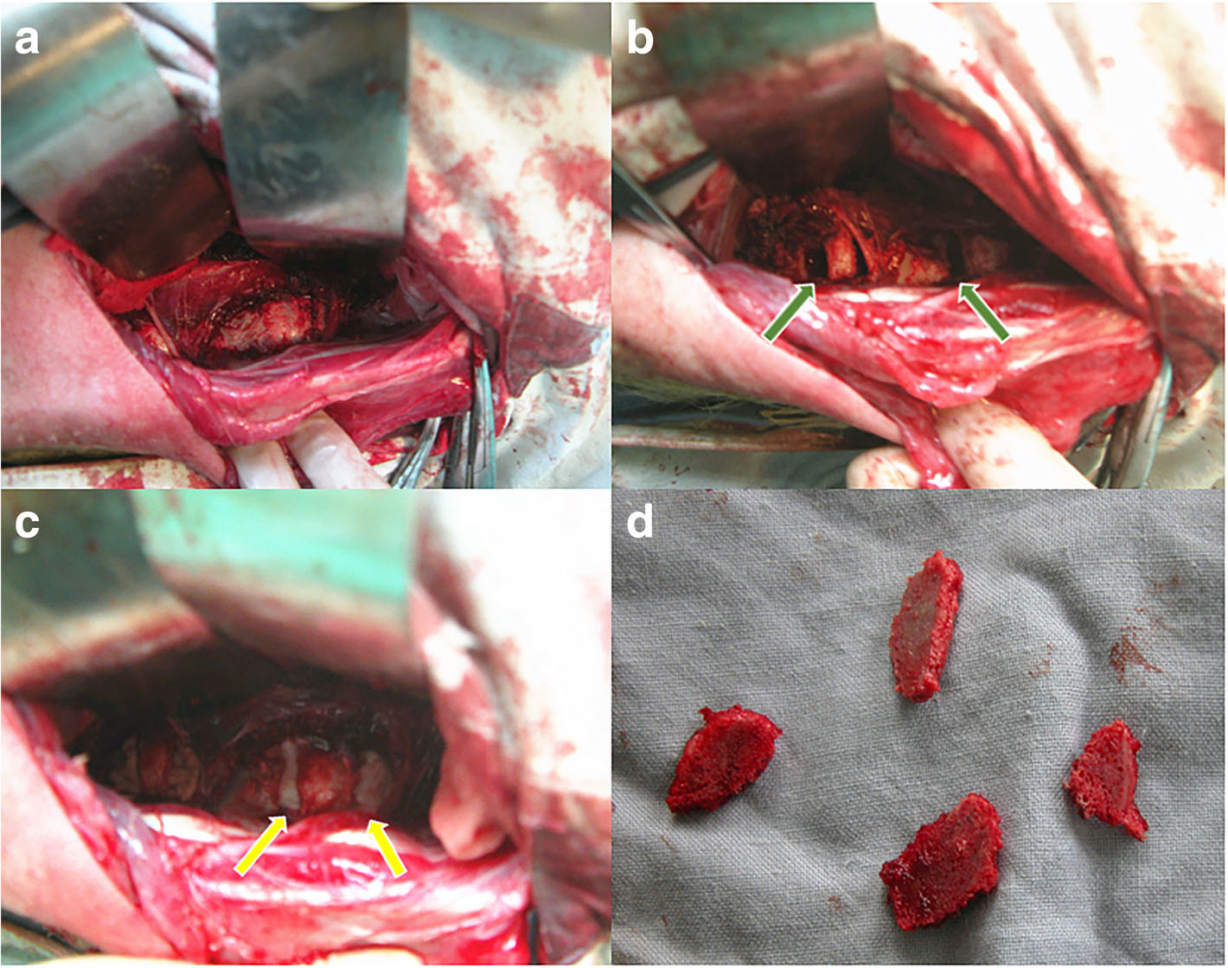
The surgical procedure of blocking both endplate nutritional pathways by cement injection. The determined lumbar vertebral bodies and intervertebral discs were exposed (**a**), and the slots were made parallel and close to the endplates (about 2 mm away) (green arrows in **b**). The bone cement was injected to fill the bone defect (yellow arrows in **c**), and the lumber vertebral laminas after osteotomy were shown in **d**

All surgeries were performed under general anesthesia in the right lateral position with adequate padding for the trunk. After routine skin preparation, sterilization, and draping, a left-sided retroperitoneal approach was used to expose the determined lumbar spine. After the skin and subcutaneous tissues were incised, abdominal wall muscular fibers (obliquus externus abdominis, obliquus internus abdominis, and transverses abdominis) were bluntly separated. After exposing the peritoneum, the posterior peritoneum was pushed away, and the psoas muscle was gently detached from the determined lumbar vertebras and intervertebral discs. Care should be taken to avoid injury of the perivertebral tissue and transverse lumbar artery intraoperatively. The discs L1–2 and L4–5 were kept intact as normal controls, while the discs L2–3 and L3–4 were exposed as the experimental targets. A slot with a width of 2 to 3 mm was made parallel and close to both the upper and lower endplates (about 2 mm away) of adjacent vertebrae to the target discs with osteotome and pendulum saw. The cancellous bone of the lumbar vertebrae was excised, while the cortical bone and endplates were retained. Then the bone cement not exceeding a maximum volume of 2 ml was used to fill the bone defect and block the endplate nutritional pathway (Fig. 1). The wound was closed in layers in an usual manner. An intramuscular injection of penicillin (1.8 million IU) was used for 3 days after surgery. The goats were housed in separate cages and fed routinely.

### Imaging evaluation

At 4, 12, 24, and 48 weeks after operation, two goats which were selected randomly and were sedated and anesthetized with Su-Mian-Xin II (intramuscular injection, 0.3 ml/kg) and propofol (intravenous drip, 2 mg/kg) for the imaging evaluation. The lateral X-ray and magnetic resonance imaging (MRI) of the lumbar spine were performed at each time point. The Philips Intera Achieva 1.5 T MRI dual-gradient system (Best, Netherlands) was used in this study. Sagittal T2-weighted imaging-turbo spin echo-spectral presaturation attenuated inversion recovery (T2WI-TSE-SPAIR) (repetition time/echo time [TR/TE] = 3500 ms/60 ms) and sagittal T1-turbo spin echo-spectral presaturation inversion recovery (T1-TSE-SPIR) (TR/TE = 400 ms/7.8 ms) were scanned, respectively. Gadobenate dimeglumine (Gd-BOPTA) which enhances contrast was injected on the goats by vein injection (a dosage of 0.3 mmol/kg) before the dynamic contrast-enhanced MRI (DCE-MRI) scan. At the time of 0 min, 5 min, 10 min, 30 min, 1.0 h, 2.0 h, and 3.0 h after the injection of the contrast media, sagittal T1-TSE-SPIR with the same variables were scanned, and lasted for 5 to 7 h in total.

The change of disc height index (DHI) was calculated as a percentage of DHI (% DHI) and normalized to the measured preoperative intervertebral disc height (% DHI = postoperative DHI/preoperative DHI × 100%). The Pfirrmann’s classification was used for disc degeneration grading from grades 1 to 5: normal (score = 1), mild degeneration (score = 2), moderate degeneration (score = 3), severe degeneration (score = 4), and advanced degeneration (score = 5) [7].

### Measurement of the effective cement blocking area

The effective blocking area was defined as the percentage of mean cement area in the vertebral body parallel to the endplate. After the discs had been excised, the bone cement was separated from the vertebrae. The accurate areas of cement and vertebral body at head and tail were calculated by using CAD software. The effective blocking area (% blocking area) was calculated as a percentage of mean cement area in the vertebral body parallel to the endplate. The specific calculation methods are as follows: % blocking area = (cranial cross-sectional area of the cement + caudal cross-sectional area of the cement)/(cranial cross-sectional area of the vertebrae + caudal cross-sectional area of the vertebrae) × 100%.

### Histology

At 4, 12, 24, and 48 weeks after operation, two goats which were selected randomly were finally euthanized after the imaging examinations. The four experimental discs (L2–3 and L3–4) and four control discs (L1–2 and L4–5) were excised at each time point. The disc specimens were fixed with 4% paraformaldehyde for at least 48 h, decalcified with 8% ethylenediaminetetra-acetate (EDTA) for 2 weeks, embedded in methyl methacrylate, and cut into slices with 4 to 6 μm thickness. Sections of discs were stained with hematoxylin-eosin (HE), Masson’s trichrome, Sirus Red, and proteoglycan stainings for photomicroscopic examinations.

### Statistical analysis

The data are reported as mean and standard deviation. One-way analysis of variance (ANOVA) test was used for the statistical analysis comparing the difference of % DHI and IDD degeneration grade between the experimental group and normal control group. Statistical significance was accepted for *P* values of < 0.05. All analyses were performed with the use of SAS software (version 9.4; SAS Institute, Cary NC, USA).

## Results

All eight goats tolerated the surgical procedure well with no major complications. The disc location of each intervention, effective cement blocking area, % DHI, and IDD grade are shown in Table 1. The mean effective blocking area percentage was 60.7 ± 5.3% (ranging from 49.6 to 69.6%). Compared with the normal control group, the discs after blocking the endplate nutritional pathways had larger changes of disc height index, 72.7 ± 5.6% versus 86.5 ± 4.6% (*P* < 0.001); and higher degrees of disc degeneration grading, 4.0 ± 0.7 versus 1.4 ± 0.5 (*P* < 0.001).

**Table 1:** The effective cement blocking area, % DHI, and IDD grade after 48 weeks of surgery

Number	Cement injection				Normal control		
	Disc location	Blocking area (%)	% DHI	IDD grade	Disc location	% DHI	IDD grade
1	L2–3	52.9	79.2	3	L1–2	85.7	2
	L3–4	57.2	75.3	4	L4–5	92.6	1
2	L2–3	54.7	81.7	3	L1–2	80.2	1
	L3–4	62.5	69.0	5	L4–5	88.0	1
3	L2–3	58.4	77.8	4	L1–2	90.5	1
	L3–4	59.8	72.7	4	L4–5	82.1	2
4	L2–3	58.5	72.6	3	L1–2	85.3	2
	L3–4	69.6	70.7	5	L4–5	93.8	1
5	L2–3	62.5	68.2	4	L1–2	84.7	1
	L3–4	67.2	60.5	5	L4–5	85.6	1
6	L2–3	63.9	66.8	4	L1–2	79.4	2
	L3–4	49.6	79.2	3	L4–5	82.0	2
7	L2–3	64.7	66.5	4	L1–2	90.5	1
	L3–4	63.2	73.8	4	L4–5	86.3	2
8	L2–3	65.7	74.0	5	L1–2	83.2	2
	L3–4	61.4	75.4	4	L4–5	93.7	1
Mean		60.7 ± 5.3	72.7 ± 5.6^*^	4.0 ± 0.7^*^		86.5 ± 4.6^*^	1.4 ± 0.5^*^

*DHI* disc height index, *IDD* intervertebral disc degeneration

^*^Difference between experimental and control groups was significant

**Fig. 2:**
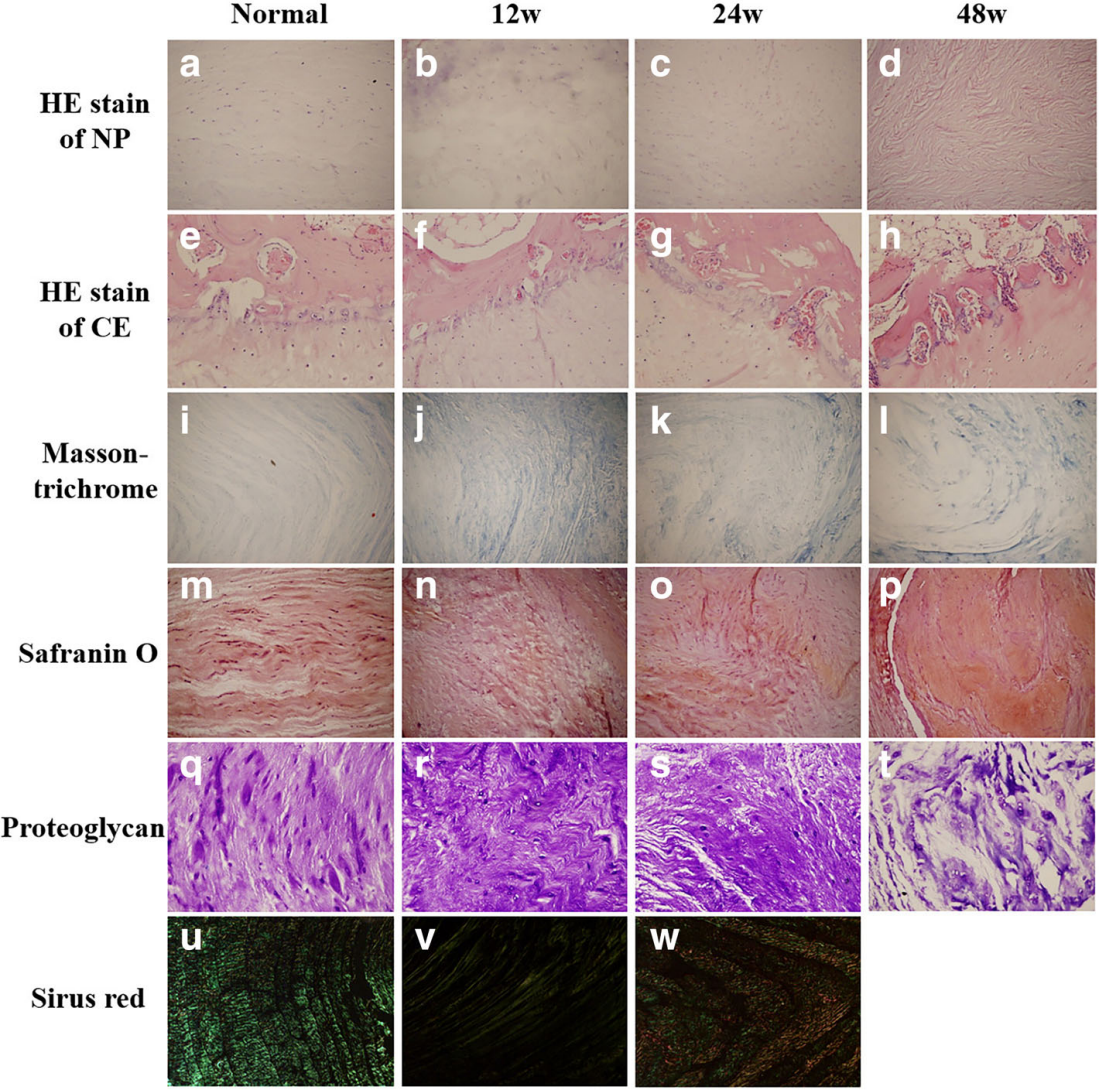
The histological images of the intervertebral discs at 12, 24, and 48 weeks after surgery. **a–d** The HE stainings of nucleus pulposus. **e–h** The HE stainings of cartilage endplates. **i–l** The Masson trichrome stainings of intervertebral discs. **m–p** The Safranin O stainings of intervertebral discs. **q–t** The proteoglycan stainings of intervertebral discs. **u–w** The Sirius Red stainings of intervertebral discs

Histological images of HE staining of NP cells and cartilage endplate (CE) are shown in Fig. 2a–h. At 12 weeks after blocking the endplate nutritional pathways, the NP showed obvious inhomogeneity of notochordal cells, cartilage-like cells, and extracellular matrix, and CE showed the disordered arrangement of cells and proliferated vessels. At 24 weeks after cement blocking, there were loss of notochordal and cartilage-like cells and increase in fibroblasts in NP, while further vascular proliferation and loss of cellular nucleus in CE were detected. At 48 weeks after operation, notochordal and cartilage-like cells in NP decreased sharply, and the CE underwent endochondral ossification, fibroblastic proliferation, and even cartilage cells disappeared. The Masson trichrome, Safranin O, and proteoglycan staining of NP also demonstrated the disruption of the uniformity of fibers, the confused alinement of fibers, and increased gaps between fibrous ring and NP in the cement blocking discs as aging of the goats (Fig. 2i–t). Other findings on histologic examination included an increase in collagen type I and a decrease in collagen type II in the cement blocking discs, which was confirmed by positive birefringent staining with Sirus Red (Fig. 2u–w). In general, the degree of degeneration of the NP and CE increased with aging of the goats.

## Discussion

Intervertebral discs, as the largest avascular organs in humans, mainly obtain nutrition by two pathways, including the endplate nutritional pathway and the annulus fibrous nutritional pathway [8]. As the most main nutritional pathway of the discs, the endplate nutritional pathway also plays important role in the degeneration of intervertebral disc [9, 10]. However, the effect of blocking the endplate nutritional pathway on the IDD was still unclear and controversial [5, 6]. Thus, the aim of this study was to explore a method for establishing stable endplate nutritional pathway inhibited animal model and investigate the relationship between the endplate nutritional pathway and IDD.

An ideal animal model of lumbar disc degeneration would have the following traits: similar anatomy and physiological characteristics compared with human beings, simulation of the development of IDD, and high repeatability [11]. We chose goats as an animal model for studying the nutritional pathway of intervertebral discs in vivo. As one kind of animal models of IDD, goats has considerable advantages such as gentle character, relatively inexpensive price, low feeding cost, good tolerance to surgery, and good simulation of developing IDD [12]. Our previous work [13] have showed that nutrient metabolism of the lumbar intervertebral discs of normal goats occurs mainly through the cartilage endplate pathway by the study of dynamic contrast enhanced MRI. On the basis of the imaging study, we established a mature goat model in which both endplate nutritional pathways were inhibited by bone cement injection.

With excellent biocompatibility and biodegradation, low immunogenicity, and a high compressive strength, polymethylmethacrylate (PMMA) cement has been widely used as a restorative material in bone defect repair and restoration [14]. The use of PMMA cement in vertebra bone defect restoration not only made it possible to efficiently block the both endplate nutritional pathways between the vertebral body and endplate, but also maintained the integrity and mechanical strength of the vertebral body, which is an obvious devoid for other blocking materials [15]. Another question is whether the heat released by polymerization reaction of PMMA cement in vertebral body could result in thermal damage of intervertebral disc and endplate, contributing to the degeneration of intervertebral disc and endplate. In a cadaveric study of vertebroplasty, Deramond et al. [16] found that the temperature at three key locations (anterior cortex, center, spinal canal) did not exceed 41 °C during polymerization of PMMA without cement leakage. Lai et al. [17] reported that when bone cement was confined within the vertebra, the temperatures at the anterior cortex, neural foramen, and posterior cortex did not rise above 45 °C, which could not directly cause thermal injury to the nearby soft tissue. Aebli et al. [18] concluded that during vertebroplasty, peak temperature in the discs did not exceed 41 °C, which could not cause thermal damage to the intervertebral discs. In the current study, both endplate nutritional pathways were blocked by bone cement injection with a width of the vertebra bone defect not exceeding 3 mm and a volume of PMMA cement not exceeding 2 ml, in order to avoid the thermal damage of intervertebral discs and endplates from polymerization of PMMA. At 4 weeks after bone cement injection, no obvious degeneration of intervertebral discs and endplates was detected by MRI or any histological examination, from which we can infer that polymerization of PMMA cement in the vertebral body could not cause the degeneration of intervertebral disc and endplate.

The nutrition supply of the nucleus pulposus is mainly dependent on the diffusion of vessels in the central area of the endplate. Urban et al. [19] found that solute transport was taken place in 85% of the bone-disc area under the nucleus and in only 35% of bone-disc area under the inner annulus, while the bone-disc interface at the outer annulus was almost completely impermeable. Oki et al. [20] carried out an experiment to examine the morphologic difference between vascular buds in two regions of the vertebral endplate in 20-week-old rabbits. The result showed that the vascular buds in the area near the nucleus pulposus exhibited swollen and complex coil-like loops with high permeability at the endplate, while those in the region of the inner annulus formed only simple loops with low permeability. In this research, the cancellous bone of lumbar vertebrae 1 to 2 mm off the endplate especially in the central area under the nucleus was excised and refilled with bone cement, in order to inhibit the nutritional pathways as much as possible. The mean effective blocking area percentage was 60.7%, which could achieve the aim of inhibiting both endplate nutritional pathways effectively in theory.

Imaging examinations at the time of 48 weeks after blocking both endplate nutritional pathways showed obvious IDD changes, including significant disc height reduction and higher disc degeneration grading compared with the control group. Several histological examinations were carried out in the current study to confirm the IDD changes after blocking both endplate nutritional pathways, including the collapse of annulus fibrosus, the loss of notochordal and cartilage-like cells, the increase in collagen type I, and the decrease in collagen type II in the cement blocking discs.

Readers should be aware of limitations in our study. We chose the adjacent discs rather than the same segments in other goats as normal controls, which may more or less influence the results of our research because of the possible adjacent segment degeneration after bone cement injection. Furthermore, there were no positive controls by needle stick injury in the discs or other means in this study.

## Conclusions

A mature goat model with both endplate nutritional pathways blocked by bone cement injection was established in the current study. The results after 48 weeks of bone cement intervention showed that the severe inhibition in the endplate nutritional pathways may result in IDD.

## Abbreviations

ANOVA: Analysis of variance
CE: Cartilage endplate
DCE-MRI: Dynamic contrast-enhanced magnetic resonance imaging
DHI: Disc height index
EDTA: Ethylenediaminetetra-acetate
Gd-BOPTA: Gadobenate dimeglumine
HE: Hematoxylin-eosin
IDD: Intervertebral disc degeneration
MRI: Magnetic resonance imaging
NP: Nucleus pulposus
NPCs: Nucleus pulposus cells
PMMA: Polymethylmethacrylate
T1-TSE-SPIR: T1-turbo spin echo-spectral presaturation inversion recovery
T2WI-TSE-SPAIR: T2-weighted imaging-turbo spin echo-spectral presaturation attenuated inversion recovery
TR/TE: repetition time/echo time

## References

[1] Kadow T, Sowa G, Vo N, Kang JD (2015). Molecular basis of intervertebral disc degeneration and herniations: what are the important translational questions?. Clin Orthop Relat Res.

[2] Zhu Q, Gao X, Levene HB, Brown MD, Gu W (2016). Influences of nutrition supply and pathways on the degenerative patterns in human intervertebral disc. Spine (Phila Pa 1976).

[3] Benneker LM, Heini PF, Alini M, Anderson SE, Ito K (2005). 2004 young investigator award winner: vertebral endplate marrow contact channel occlusions and intervertebral disc degeneration. Spine (Phila Pa 1976).

[4] Rajasekaran S, Venkatadass K, Naresh Babu J, Ganesh K, Shetty AP (2008). Pharmacological enhancement of disc diffusion and differentiation of healthy, ageing and degenerated discs: results from in-vivo serial post-contrast MRI studies in 365 human lumbar discs. Eur Spine J.

[5] Hutton WC, Murakami H, Li J, Elmer WA, Yoon ST, Minamide A (2004). The effect of blocking a nutritional pathway to the intervertebral disc in the dog model. J Spinal Disord Tech.

[6] Kang R, Li H, Ringgaard S, Rickers K, Sun H, Chen M (2014). Interference in the endplate nutritional pathway causes intervertebral disc degeneration in an immature porcine model. Int Orthop.

[7] Pfirrmann CW, Metzdorf A, Zanetti M, Hodler J, Boos N (2001). Magnetic resonance classification of lumbar intervertebral disc degeneration. Spine (Phila Pa 1976).

[8] Wu Y, Cisewski SE, Wegner N, Zhao S, Pellegrini VD, Slate EH (2016). Region and strain-dependent diffusivities of glucose and lactate in healthy human cartilage endplate. J Biomech.

[9] Urban JP, Smith S, Fairbank JC (2004). Nutrition of the intervertebral disc. Spine (Phila pa 1976).

[10] Hebelka H, Miron A, Kasperska I, Brisby H, Lagerstrand K (2018). Axial loading during MRI induces significant T2 value changes in vertebral endplates-a feasibility study on patients with low back pain. J Orthop Surg Res.

[11] Dowdell J, Erwin M, Choma T, Vaccaro A, latridis J, Cho SK (2017). Intervertebral disk degeneration and repair. Neurosurgery.

[12] Daly C, Ghosh P, Jenkin G, Oehme D, Goldschlager T (2016). A review of animal models of intervertebral disc degeneration: pathophysiology, regeneration, and translation to the clinic. Biomed Res Int.

[13] Du H, Ma SH, Guan M, Han B, Yang GF, Zhang M (2011). Dynamic contrast enhanced-magnetic resonance imaging study of the nutrition pathway for lumbar intervertebral disk cartilage of normal goats. Orthop Surg.

[14] Robinson Y, Heyde CE, Försth P, Olerud C (2011). Kyphoplasty in osteoporotic vertebrae compression fractures guidelines and technical considerations. J Orthop Surg Res.

[15] Höch A, Schimpf R, Hammer N, Schleifenbaum S, Werner M, Josten C (2017). Biomechanical analysis of stiffness and fracture displacement after using PMMA-augmented sacroiliac screw fixation for sacrum fractures. Biomed Tech (Berl).

[16] Deramond H, Wright NT, Belkoff SM (1999). Temperature elevation caused by bone cement polymerization during vertebroplasty. Bone.

[17] Lai PL, Tai CL, Chen LH, Nien NY (2011). Cement leakage causes potential thermal injury in vertebroplasty. BMC Musculoskelet Disord.

[18] Aebli N, Goss BG, Thorpe P, Williams R, Krebs J (2006). In vivo temperature profile of intervertebral discs and vertebral endplates during vertebroplasty: an experimental study in sheep. Spine (Phila Pa 1976).

[19] Urban JP, Holm S, Maroudas A, Nachemson A (1977). Nutrition of the intervertebral disk. An in vivo study of solute transport. Clin Orthop Relat Res.

[20] Oki S, Matsuda Y, Shibata T, Okumura H, Desaki J (1996). Morphologic differences of the vascular buds in the vertebral endplate: scanning electron microscopic study. Spine (Phila Pa 1976).

